# Evolutionary Trends of the Transposase-Encoding Open Reading Frames A and B (*orfA* and *orfB*) of the Mycobacterial IS*6110* Insertion Sequence

**DOI:** 10.1371/journal.pone.0130161

**Published:** 2015-06-18

**Authors:** Sara Thabet, Amine Namouchi, Helmi Mardassi

**Affiliations:** Unit of Typing and Genetics of Mycobacteria, Laboratory of Molecular Microbiology, Vaccinology, and Biotechnology Development; Institut Pasteur de Tunis, Tunis, Tunisia; University of Padova, Medical School, ITALY

## Abstract

**Background:**

The IS*6110* insertion sequence, a member of the IS3 family of insertion sequences, was found to be specific to the *Mycobacterium tuberculosis* complex (MTBC). Although IS*6110* has been extensively characterized as a transposable genetic marker, the evolutionary history of its own transposase-encoding sequence has not, to the best of our knowledge, been investigated.

**Methodology/Principal Findings:**

Here we explored the evolution of the IS*6110* sequence by analysing the genetic variability and the selective forces acting on its transposase-encoding open reading frames (ORFs) A and B (*orfA* and *orfB*). For this purpose, we used a strain collection consisting of smooth tubercle bacilli (STB), an early branching lineage of the MTBC, and present-day *M*. *tuberculosis* strains representing the full breadth of genetic diversity in Tunisia. In each ORF, we found a major haplotype that dominated over a flat distribution of rare descendent haplotypes, consisting mainly of single- and double-nucleotide variant singletons. The predominant haplotypes consisted of both ancestral and present-day strains, suggesting that IS*6110* acquisition predated the emergence of the MTBC. There was no evidence of recombination and both ORFs were subjected to strict purifying selection, as demonstrated by their dN/dS ratios (0.29 and 0.51, respectively), as well as their significantly negative Tajima’s *D* statistics. Strikingly, the purifying selection acting on *orfA* proved much more stringent, suggesting its critical role in regulating the transpositional process. Maximum likelihood analyses further excluded any possibility of positive selection acting on single amino acid residues.

**Conclusions/Significance:**

Taken together our data fit with an evolutionary scenario according to which the observed variability pattern of the IS*6110* transposase-encoding ORFs is generated mainly through random point mutations that accrued on a functionally optimal IS*6110* copy, whose acquisition predated the emergence of the MTBC complex. Background selection acting against deleterious mutations led to an excess of low-frequency variants.

## Introduction

Insertion sequences (ISs) are the smallest autonomously transposable mobile genetic elements widely distributed in bacterial genomes [1–4]. IS elements carry in their sequence the genes encoding for a transposase, thus ensuring their mobility in the bacterial genome. IS mobilization and expansion via transposition contribute significantly to genome diversification and plasticity [4–6]. Indeed, IS sequences have been shown to induce mutations, duplications, deletions, inversions, as well as complex genomic rearrangements [4,7]. In many circumstances, insertion of IS elements modulates the expression of neighbouring genes, which may benefit the organism, thus arguing for a consistent role in adaptive evolution, particularly for those bacteria adopting fastidious, host-restricted lifestyles [1,8,9,10,11,12].

*Mycobacterium tuberculosis*, a highly adapted and host-restricted pathogen, carries in its genome at least 30 different IS elements, one of which, IS*6110*, is found exclusively in members of the *M*. *tuberculosis* complex (MTBC) [13–15]. The latter species comprises *Mycobacterium tuberculosis*, *Mycobacterium bovis*, *Mycobacterium microti*, *Mycobacterium africanum*, *Mycobacterium pinnipedii* and *Mycobacterium caprae* [15,16]. Strong evidence suggests that the MTBC group has arisen from a pool of mycobacterial species, referred to as smooth tubercle bacilli (STB), including the species “*Mycobacterium canettii*” [17–21]. IS*6110* was originally identified in 1990 and proved very useful as a marker for the molecular detection of MTBC strains [22–31]. The highly polymorphic nature of IS*6110* in terms of copy number and transpositional location has provided the most widely used fingerprinting approach, IS*6110* RFLP. Owing to this typing method, it has been possible to distinguish recent transmission from reactivation, to uncover TB transmission dynamics, and to delineate the population structure of *M*. *tuberculosis* in diverse settings worldwide [32–34].

IS*6110* belongs to the IS3 family of insertion sequences [24]. It consists of a 1361-bp sequence with imperfect 28 bp terminal inverted repeats, whose transposition generates a 3–4 bp duplication at the insertion point. The IS*6110* sequence contains two partially overlapping reading frames (ORFs), *orfA* and *orfB*. Based on sequence similarities with the IS3 element of *E*. *Coli* [35], it is assumed that a -1 translational frameshifting between *orfA* and *orfB* would generate an *orfAB* transframe protein, the IS*6110* transposase [34]. The sequence “UUUUAAAG” located directly upstream of the IS*6110 orfB* may be responsible for such a frameshifting event [24]. Like IS3 [33–35], IS*6110 orfA* protein contains a helix-turn-helix DNA-binding domain (residues 16 to 58), while the *orfB* protein displays characteristics reminiscent of retrovirus and retrotransposon integrases [36–38].

Compelling evidence suggests that variations in IS*6110* transposition induce new strain-specific phenotypic changes, either by mediating genomic rearrangements, or by acting as a promoter sequence that modulates the expression of neighbouring genes [39–46]. Hence, IS*6110* is no more viewed as a passive ‘junk” or “selfish parasite” DNA sequence, but as a significant contributor to the evolution of *M*. *tuberculosis* [34].

To date, IS*6110* has been extensively studied as a transposable element that induces several mutational changes on the host chromosomal DNA. Despite the fact that the transposition process of IS*6110* is mediated by its own transposase, a very few studies have focused on the transposase-encoding sequences. In the present study we sought to explore the genetic variability of the IS*6110* transposase-encoding ORFs, and determine the selective pressures acting on their coding sequences.

## Materials and Methods

### Ethics statement

This investigation involved only DNA from Mycobacteria that have previously been described and published. No sputum or any other samples were collected from patients for the specific needs of this study.

### *M*. *tuberculosis* strains

This study was performed using 63*M*. *tuberculosis* (MTB1 to MTB63) clinical strains representing diverse genotypes circulating in Tunisia between 2001 and 2005. This clinical strain collection consisted of the following spoligotypes: 1 Latin American Mediterranean 1 (LAM1) (ST20), 4 LAM4 (ST60), 1 LAM4 (ST828), 2 LAM5 (ST93), 12 LAM9 (ST42), 2 LAM9 (ST177), 1 LAM9 (ST398), 2 LAM9 (ST822), 2 LAM9 (ST1064), 6 Haarlem 1 (H1) (ST47), 1 H1 (ST883), 1 Haarlem 3 (H3) (ST49), 7 H3 (ST50), 1 H3 (ST56), 1 H3 (ST180), 1 H3 (ST871), 1 H3 (ST121), 1 H3 (ST764), 1T1 (ST7), 10 T1 (ST53), 1 T1 (ST281), 1 T2 (ST52), 1 MANU2 (ST54), 1 S (ST34), and 1 S (ST1536). Assignment of these genotypes was performed based on the SITVITWEB database (http://www.pasteur-guadeloupe.fr:8081/SITVIT_ONLINE/). We also included 20 smooth tubercle bacilli (STB1 to STB20) covering 6 genotypes: B (STB1), C/D (STB2-STB15), E (STB16), F (STB17), G (STB18), and H (STB19-STB20). These STB strains have been described earlier [19,20]. Details regarding the origin and year of isolation of the mycobacterial isolates are listed in S1 Table.

### PCR amplification and cloning

DNA extraction was performed by the phenol chloroform method [23]. The primer pair IS*6110*F (5’-ATCTGAACCGCCCCGGCATGTCCGG-3’) and IS*6110*R (5’-ATCTGAACCGCCCCGGTGAGTCCGG-3’) was used for PCR amplification of the IS*6110* sequence. The amplification reaction mixture contained 20 ng of template genomic DNA, 10 μl of 10x buffer (Qiagen), 10 μl DMSO, 2 μl of 10 mM nucleotide mix (Amersham Biosciences), 2 μl of each primer (20 μM stock), 0.25 μl (1.25 U) of HotStar *Taq* DNA polymerase (Qiagen) and sterile nuclease-free water (Amersham Biosciences) to 50 μl total reaction volume. Cycling was carried out in a 2720 thermocycler (Applied Biosystems) with an initial denaturation step of 10 min at 96°C followed by 35 cycles consisting of 1 min at 95°C, 1 min at 60°C and 2 min at 72°C. The amplification ended with a final elongation step of 7 min at 72°C.

One to 2 μL of each PCR product was directly cloned into the pCR2.1 plasmid vector using the TA cloning kit, following the manufacturer’s instructions (Life Technologies). The ligation product was then transformed into chemically competent TOP10 *E*. *coli*, and 200 μL were plated for blue/white screening using LB agar plates containing 40 μl of X-Gal (40 mg/mL) and ampicilin (100 μg/mL). Ten white colonies from each ligation were allowed to grow overnight in 2YT (containing 100 μg/mL amplicillin), then plasmid DNA was recovered using the QIAprep spin miniprep kit (Qiagen). Recombinant clones were confirmed by *EcoR*I digestion.

### Sequencing

A single IS*6110* copy from each mycobacterial strain was subjected to nucleotide sequencing on both strands of the recombinant pCR2.1 plasmid using the M13 reverse and forward primers. Sequencing was performed with the BigDye Terminator Cycle Sequencing Kit. The reaction consisted of 1.5 μl of BigDye terminator cycle sequencing reagents, 4 μl of BigDye terminator cycle sequencing buffer, 1 μl of 20 μM concentrations of primers, and UltraPURE Distilled DNase/RNase-FreeWater (Gibco/Invitrogen) to make a 20-μl reaction. Cycle sequencing was performed using a 2720 thermocycler (Applied Biosystems) programmed for 25 cycles at 96°C for 10 s, 50°Cfor 5 s, and 60°C for 4 min. The template DNA was ethanol-precipitated, washed, and subjected to automated sequencing on an ABI Prism 3130 genetic analyzer (Applied Biosystems) according to the manufacturer's protocol.

### Genetic polymorphism and diversity

Sequence data of the 83 IS*6110* copies were edited and aligned using BioEdit (https://www.bioedit.com/) and ClustalW [47]. The DnaSP software package (version.5.10) [48] was used to carry out several population genetic analyses. For both IS*6110 orfA* and *orfB*, we determined the number of haplotypes (h), the number of polymorphic sites (S), the nucleotide diversity (π), and the per-site population mutation rate, θ (2N_e_μ). To test for adaptive selection, we determined the nucleotide substitution changes and the ratio of nonsynonymous (dN) to synonymous (dS) substitutions per site (dN/dS), using the analysis developed by Nei-Gojobori [49] after Jukes-Cantor correction for multiple substitutions.

### Phylogenetic analysis

Maximum likelihood (ML) methods were used to infer phylogenetic relationships. ML analyses were performed usingPhyML 3.0 [50]. Bootstrap confidence levels were based on 1000 resampling. The trees were visualized using FigTree (http://tree.bio.ed.ac.uk/software/figtree/).

Prior to ML analyses, we determined for each dataset the best-fit model of nucleotide substitution (see S2 Table) using jModelTest 2 [51].

To test for the topological congruence between trees, we computed the *Icong* index, which is based on maximum agreement subtrees (MAST) [52]. This method determines the minimum number of leaves that have to be removed in each phylogeny to render the trees identical. Computation of *Icong* and of the associated *P*-value was performed on line at http://max2.ese.u-psud.fr/bases/upresa/pages/devienne.

### Tests of recombination

As a first indication of the eventual existence of recombination, we performed a split decomposition analysis to generate phylogenetic networks [53]. The networks were generated in Splitstree 4.6 [54]. Evidence of recombination, indicated by the presence of cycles in the networks, was assessed by the pairwise homoplasy index (PHI). Significance of the PHI statistic is assessed with the normal approximation of a permutation test where, under the null hypothesis of no recombination, sites along the alignment are randomly permuted to obtain the null distribution of PHI. *P* values < 0.05 indicate significant presence of recombination [55]. To confirm the results obtained with the split decomposition analysis, we used two additional recombination detection algorithms:

- Hudson and Kaplan’s *R*_min_ [56]: The Hudson and Kaplan’s lower bound on the minimal number of recombination events in an infinite site model was computed usingthe DnaSP software package (version.5.10) [48].
- The Web-based service GARD (genetic algorithm for recombination detection) [57]: a model-based approach that searches for putative breakpoints delimiting sequence regions having distinct phylogenies. Briefly, GARD compares a nonrecombinant model in which the sequence data are fitted to a single phylogeny to models in which breakpoints partition the sequence data into two or more regions having varying phylogenies. Site-by-site substitution rate was assumed to be constant between sites. The identified breakpoints were further confirmed using the akaike information criterion (AIC) score and Kishino-Hasegawa topological incongruence test.

### Tests of selection

To search for signals of positive selection acting on IS*6110* ORFs, we first measured the ratio of nonsynonymous substitutions to synonymous substitutions, dN/dS ratio (or *ω*), over the entire length of each ORF. Evidence of positive selection for amino acid replacements is suggested when *ω*> 1 (adaptive evolution; nonsynonymous changes are favored because they confer a fitness advantage and are fixed at a higher rate than synonymous changes), purifying selection is inferred when *ω*< 1 (strong functional constraint; nonsynonymous changes are deleterious for protein function and are fixed at a lower rate than synonymous changes), whereas neutral evolution is assumed when *ω* = 1 (relaxed selective constraint; nonsynonymous changes have no associated fitness advantage and are fixed at the same rate as synonymous changes). Then we performed a codon-by-codon analysis using codeml as implemented in the software package PAML (Phylogenetic Analysis by Maximum Likelihood) v. 4.4e [58]. For this purpose we used ‘‘site models” where codon sites are allowed to fall into categories depending on their *ω* values. First, we compared a “nearly neutral model”, M1a, to a “positive selection” model, M2a. The model M1a allows 2 categories of codon sites in *p*_0_, and *p*_1_ proportions, with *ω*_0_<1 and, *ω*_1_ = 1, whereas M2a adds an additional category of codons (*p*_2_), with *ω*_2_ that is free to vary above 1. In addition to M1a and M2a, we compared several additional site models, M7, M8, and M8a. M7 specifies a neutral model similar to M1a, but the sites affected by negative selection approximate a beta distribution with parameters (*p* and *q*) estimated from the data. M7 is compared to M8 (selection) for which the category of sites with a dN/dS > 1 is added. We also compared the model M8 to M8a. In the latter model the extra *ω* is fixed at 1. Previous studies have shown that the M8-M8a comparison is more robust than the M7-M8 comparison and produces less false positives [59,60].

The comparison between models was assessed using Likelihood-Ratio Tests (LRTs). A significantly higher likelihood of the alternative model than that of the null model indicates positive selection in the data set examined. For models comparisons, we used degree of freedom, df = 2. For each analysis, correction for multiple testing (Bonferroni correction) was applied. Only in cases where LRT was significant, we used the Bayes empirical Bayes (BEB) procedure to calculate the posterior probabilities (PPs) to identify sites under positive selection [61].

### Nucleotide sequence accession numbers

IS*6110* ORFs A and B haplotype nucleotide sequences were deposited in GenBank under accession numbers KP844666 to KP844685 and KP844686 to KP844721, respectively.

## Results

### Sequence diversity of IS*6110* transposase-encoded ORFs A and B

**Fig 1:**
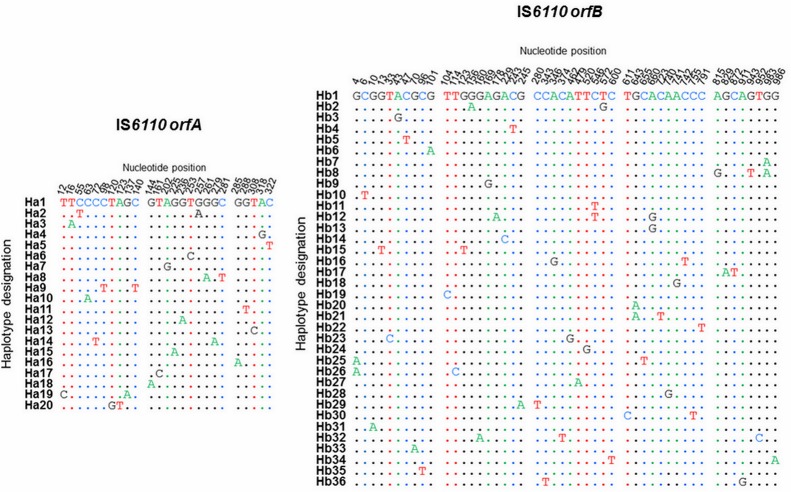
Sequence alignment of IS*6110 orfA* and *orfB* haplotypes.

To appreciate the inter-strain genetic diversity of IS*6110* transposase-encoded *orfA* and *orfB*, we sequenced a single IS*6110* copy from each strain of STB and *M*. *tuberculosis*. The statistics are reported in Table 1. Overall, the *orfA* showed slightly higher nucleotide diversity (π) compared to *orfB* (0.00199 vs 0.00132), as well as a higher population mutation rate (θ) (0.01532 vs 0.00976). As expected, much of the nucleotide diversity in *orfA* was associated with the STB group, which is three fold higher than in today’s *M*. *tuberculosis* strains (0.00398 vs 0.00135). In both ORFs, mutations were distributed throughout the entire sequence (Fig 1). Only in one case (strain MTB20), did a nonsense mutation occurred, yielding an *orfB* product lacking the 13 C-terminal last residues.

**Table 1:** Patterns of molecular genetic diversity of IS*6110 orfA* and *orfB*.

	No. of polymorphic sites (S)	No. of Haplotypes (h)	Haplotype diversity (Hd)	Nucleotide diversity (π)	per-site population mutation rate (θ)	No. of nsSNPs	No. of sSNPs	dN/dS (*ω*)	Tajima test statistic (*D)*
***orfA* (STB)**	13	11	0.763	0.00398	0.01121	8	5	0.58743	-2.34049*
***orfA* (MTB)**	12	10	0.2939	0.00135	0.00779	4	8	0.14906	-2.35117*
***orfB* (STB)**	15	12	0.8105	0.00152	0.00428	9	6	0.39413	-2.38105*
***orfB* (MTB)**	36	25	0.6395	0.00125	0.00775	24	11	0.58168	-2.74469**
***orfA* (STB + MTB)**	25	20	0.4258	0.00199	0.01532	12	13	0.29613	-2.65444**
***orfB* (STB + MTB)**	48	36	0.6820	0.00132	0.00976	32	15	0.51483	-2.79601**

*P< 0.01

**P< 0.001

**Fig 2:**
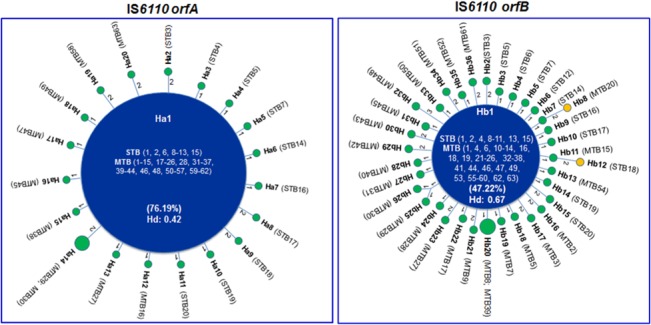
Parsimonious relationships among haplotypes of IS*6110 orfA* and *orfB*. Each IS*6110* transposase-encoding haplotype is represented with a filled circlewhose size reflects its frequency. The predominant haplotype is shown in blue, while the descendent rare haplotypes are shown in green and orange. Haplotype designations (Ha1 to Ha20 or Hb1 to Hb36 for *orfA* and *orfB*, respectively) and their associated mycobacterial strains are indicated within or next to the circles. The number of mutations dividing the haplotypes is shown next to the lines connecting the circles. Frequency of the predominant haplotypein each ORF is indicated within parenthesis. Hd: haplotype diversity.

Twenty and 36 haplotypes were identified for IS*6110* ORFs A and B (Ha1 to Ha20 and Hb1 to Hb36). Both ORFs displayed low haplotype diversity (0.42 vs 0.68) and showed similar haplotype distribution patterns. Indeed, in each ORF we observed a predominant haplotype, involving 76.19% and 47.22% of the entire strain collection in *orfA* and *orfB*, respectively (Fig 2). These frequent haplotypes dominate over a flat distribution of rare descendent haplotypes that differ mainly by a single or two mutations. In both ORFs, the predominant haplotype included ancestral (STB) and present-day (MTB) strains (Fig 2). It is noteworthy that *orfA* and *orfB* sequences of an IS*6110* copy of the reference strain H37Rv [13], each belongs to the respective predominant haplotype.

Tajima’s *D* statistics proved significantly negative for both ORFs (Table 1),which is consistent with their observed haplotype distribution pattern that is characterized by an excess of rare variants (singletons).

Furthermore, both *orfA* and *orfB* evolved under strict purifying selection, since their dN/dS values are < 1 (0.29 and 0.51, respectively). The *orfA* is likely to be the subject of more stringent purifying action compared to *orfB* (Table 1). Indeed, in *orfB* the ratio nsSNP/sSNP is 2.13 (32/15), which is in the range known for *M*. *tuberculosis* genome, while this ratio is about 0.92 for *orfA*, suggesting that non synonymous changes tend to be purged during its evolution.

### Evaluation of the congruence of *orfA* and *orfB* phylogenies

**Fig 3:**
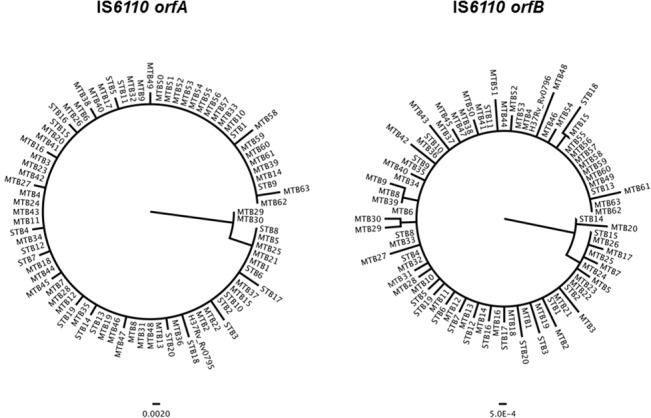
ML Phylogenetic trees showing the relationships of STB and MTB strains based on the IS*6110* transposase-encoding *orfA* and *orfB*.

To better appreciate the evolutionary trends of both IS*6110* transposase-encoded ORFs, we constructed their ML trees and assessed their congruence. The ML trees, shown in a polar format, are depicted in Fig 3. Consistent with the haplotype diversity patterns of both ORFs, the tree topography shows virtually no deep branching; the majority of tips being nearly at equivalent genetic distances.

When the strain collection was analyzed as a whole (STB + MTB), the phylogenies of *orfA* and *orfB* were found congruent (*Icong* = 3.121; *P* = 2.045e^-20^). However, a conflict between *orfA* and *orfB* trees was detected in the STB group (*Icong* = 1.199; *P* = 0.083), but not in today’s MTB strains (*Icong* = 2.898; *P* = 6.474e^-19^).

### Lack of evidence for recombination

**Fig 4:**
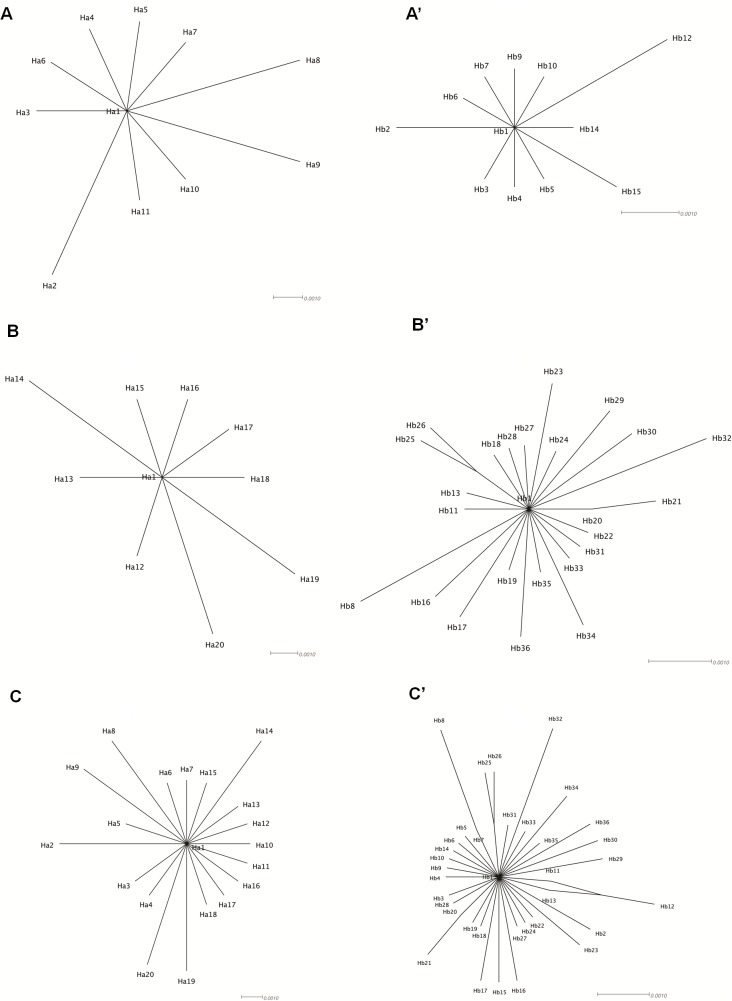
Split decomposition analysis based on IS*6110*ORFs A and B haplotype sequences. Panels A, C, and D represent the *orfA*-based phylogenetic networks of STB, MTB, and the whole collection (STB + MTB), respectively. Panels A’, B’, and C’ depict the *orfB*-based phylogenetic networks of STB, MTB, and the whole collection (STB + MTB), respectively.

As a repeat sequence, IS*6110* could be involved in recombination-mediated genomic rearrangement events. Therefore, we searched for signals of recombination, and for this purpose, we first performed a split decomposition analysis. The tree topography (Fig 4) and the statistical PHI test did not provide evidence for recombination. This finding was further confirmed with two additional approaches, the Hudson and Kaplan’s *R*_min_ and GARD, which found no evidence for breakpoints.

### IS*6110* transposase-encoding *orfA* and *orfB* evolved under strict purifying selection

Despite the fact that both IS*6110 orfA* and *orfB* were clearly evolving under purifying selection, as suggested by their dN/dS ratios and their negative Tajima’s *D* statistic, one should not dismiss the possibility that positive selection could have operated on specific codons. For this purpose, we performed a codon-by-codon maximum likelihood test using the program codeml (PAML package). As shown in Table 2, in no case was the difference of the likelihood ratio test significant for both models comparisons (M1a vs M2a and M8a vs M8), indicating absence of positive selection acting at specific codons.

**Table 2:** Likelihood ratio test (LRT) analysis of models comparisons M1a vs M2a and M8 vs M8a.

	M1a	M2a	M8	M8a
***orfA* (MTB)**	-516.184909	-514.152318	-514.155047	-514.155030
**LRT *p-value***	0.13		0.999984	
***orfA* (STB)**	-514.941268	-514.941267	-514.942165	-514.942159
**LRT *p-value***	1		0.999995	
***orfA* (MTB+STB)**	-611.532742	-611.532720	-611.541343	-611.540493
**LRT *p-value***	0.999979		0.999151	
***orfB* (MTB)**	-1531.882528	-1531.882524	-1532.269623	-1531.885179
**LRT *p-value***	0.999997		0.68083	
***orfB* (STB)**	-1400.206582	-1400.206579	-1400.208005	-1400.207932
**LRT *p-value***	0.999998		0.999928	
***orfB* (MTB-STB)**	-1659.837451	-1659.837445	-1659.840249	-1659.840053
**LRT *p-value***	0.999995		0.999805	

## Discussion

In the present study we explored the genetic variability and the selective forces acting on the IS*6110* transposase-encoding ORFs, *orfA* and *orfB*. For this purpose, we used a strain collection consisting of smooth tubercle bacilli (STB), an early branching lineage of the MTBC, and present-day *M*. *tuberculosis* strains representing the full breadth of genetic diversity in Tunisia. Based on the roles of the *E*. *Coli* IS3 insertion sequence [62], it has been suggested that IS*6110 orfA* and *orfB* products may act as negative regulators to limit the number of IS*6110* transposition events, thereby minimizing an eventual deleterious effect to the mycobacterial host genome [34]. By testing several models of transposition Tanaka et al. [63] provided evidence for selection against IS*6110* in *M*. *tuberculosis*, a finding in line with the possible roles assigned to the products of *orfA* and *orfB*.

Taken together, our data converge towards an evolutionary scenario according to which IS6110 had been acquired early before emergence of the MTBC. This conclusion stems from the fact that for both ORFs a single haplotype predominated, consisting of admixtures of ancestral and present-day strains. Early acquisition of IS*6110* is also suggested by the fact that all strains of the ancestral STB pool tested thus far contain the insertion element IS*6110*, the copy number of which varies from 1 (groups E and F) to 10 (groups G an H) [20]. Hence, the predominant haplotype identified herein, which is common to both STB and present-day *M*. *tuberculosis* strains, could represent the very early copy inherited from the common ancestor of the STB population. The ancestral origin of IS*6110*, as confirmed in the present study, is consistent with the identification of IS*6110*-like sequences from environmental mycobacterial species (*M*. *smegmatis*, *Mycobacterium* sp. JLS) [64], thus lending further support to the hypothesis that the ancestor of the MTBC could have originated from an environmental mycobacteria [21].

Increased sequence variability of IS*6110* ORFs was observed among STB strains compared to *M*. *tuberculosis*. This finding was somehow expected based on previously published data with other sequence categories, such as houses keeping genes [19,20], PE_PGRS [65], as well as whole genome comparisons of selected STB genomes [21].

Evolution of IS*6110 orfA* and *orfB* appears to have been driven mainly by random point mutations rather than recombination, as witnessed by the distribution pattern of mutations along the two ORF sequences. We could demonstrate no signal of recombination despite the fact that several deletions in *M*. *tuberculosis* were shown to have resulted from recombinational events between two adjacent IS*6110* copies [39,41,42]. However, these events should have involved nearly identical IS*6110* sequence copies and therefore no imprints of recombination are left.

The genealogies of *orfA* and *orfB* were congruent in today’s *M*. *tuberculosis* strains, a finding in line with their cooperative role in regulating transpositional recombination [62]. By contrast, a conflict between the two genealogies was observed in STB, the underlying mechanism of which remains to be determined in the absence of demonstrable recombination.

Both IS*6110* ORFs were found to evolve under strict purifying selection. Indeed, the majority of variants consisted of singletons that differed mainly by one or two mutations, with a clear tendency to purge non synonymous changes (dN/dS < 1). Furthermore, PAML analysis could not detect any positively selected residue along the amino acid sequence of both ORFs. Given the ancestral origin of IS*6110*, these findings strongly suggest that the MTBC ancestor could have acquired, from the outset, a functionally optimal IS*6110* copy that does no more tolerate further amino acid changes. Therefore, most of the non synonymous changes that were detected are likely to be neutral or slightly deleterious [66].

The low dN/dS rate ratios in several bacterial transposase genes may not only be linked to purifying selection, but could also result from periodic extinction events of IS elements followed by the acquisition of evolutionarily young copies [67]. In Wolbachia, the low nucleotide divergence rates of IS sequences has been proposed to be due to recent import of IS sequences, via horizontal gene transfer, coupled to subsequent bursts of transposition [68]. These mechanisms could not have accounted in the evolution of the IS*6110* transposase in MTBC species, since they have undergone a clonal evolution with virtually no genetic exchanges, thus pointing to the prominent role of purifying selection.

However, positive selection was shown to act on IS transposases of other bacterial species, such as *E*. *coli* and the cyanobacterium *Crocosphaera watsonii* [69,70]. In *E*. *coli*, positive selection was found to operate on the IS30 and IS1 transposase genes. In IS30, evidence of positive selection could be detected in 16 sites, of which 14 occur in the N-terminal helix-turn-helix motif 1 (HTH1), thus favoring particular sites to be frequently targeted by transposition. As far as could be ascertained, IS*6110* does not have a known target for insertion, and despite the existence of insertion hotspots, it tends to integrate the mycobacterial genome at random [71–77]. This is consistent with the absence of positive selection acting on its transposase sequence. However, one should not dismiss the possibility that the IS*6110* transposase sequence could have been the subject of positive selection early in its evolutionary history.

In line with its critical role in IS*6110* transpositional control, the *orfA* was found to be the subject of more stringent purifying selection compared to *orfB* (nsSNP/sSNP: 0.92 vs 2.13). Indeed, in the IS3 element of *E*. *coli*, both ORFs were shown to act as inhibitors of transpositional recombination. It has been demonstrated that such inhibition could be mediated by the *orfA* product alone, while *orfB* by itself has no inhibitory activity, but enhances the inhibitory activity of *orfA*, probably by interacting with *orfA* to form a complex. In other words, *orfB* exerts its negative effect on transpositional recombination only when *orfA* is produced, and thus functions in cooperation with *orfA* [62]. Consequently, for an optimal control of IS*6110* transposition activity, background selection should act more frequently on *orfA*, which we demonstrate herein.

## Conclusions

The haplotype distribution characterizing the mycobacterial IS*6110* transposase-encoding ORFs Aand B strongly suggests its ancestral origin, which most likely predated emergence of the MTBC. These ORFs evolved essentially by point mutations under strict purifying selection acting against deleterious mutations, thus leading to an excess of low-frequency variants. Aside from purging non synonymous changes, no imprints of positive selection acting on single amino acid residues could be detected in both STB and present-day *M*. *tuberculosis* strains, arguing that the IS*6110* copy they had inherited was functionally optimal. Finally, the fact that *orfA* was the subject of more stringent purifying selection compared to *orfB*, lends further support to its essential role in regulating the IS*6110* transpositional process.

## Supporting Information

S1 Table Characteristics of STB and *M*. *tuberculosis* strain collections used in this study. (DOCX)

S2 Table The best-fit model of nucleotide substitution as determined by jModelTest 2 [50]. (DOCX)
