# Supplementary material for: Evolutionary Trends of the Transposase-Encoding Open Reading Frames A and B (orfA and orfB) of the Mycobacterial IS6110 Insertion Sequence
Source: PLoS One. 2015 Jun 18;10(6):e0130161. doi: 10.1371/journal.pone.0130161 (PMC4473070; doi:10.1371/journal.pone.0130161)
Supplement: S1 Table — (DOCX) [file pone.0130161.s001.docx]

S1 Table. Characteristics of STB and *M. tuberculosis* strain collections used in this study.

| Strain | Species | Year of isolation | Genetic group^1^ or spoligotype^2^ | Shared type^2^ |
| --- | --- | --- | --- | --- |
| STB1 | STB | 1999 | B | - |
| STB2 | STB | 1999 | C/D | - |
| STB3 | STB | 1999 | C/D | - |
| STB4 | STB | 1999 | C/D | - |
| STB5 | STB | 1999 | C/D | - |
| STB6 | STB | 1999 | C/D | - |
| STB7 | STB | 1999 | C/D | - |
| STB8 | STB | 2000 | C/D | - |
| STB9 | STB | 2000 | C/D | - |
| STB10 | STB | 2000 | C/D | - |
| STB11 | STB | 2002 | C/D | - |
| STB12 | STB | 2002 | C/D | - |
| STB13 | STB | 2002 | C/D | - |
| STB14 | STB | 2003 | C/D | - |
| STB15 | STB | 2003 | C/D | - |
| STB16 | STB | 1999 | E | - |
| STB17 | STB | 1997 | F | - |
| STB18 | STB | 2000 | G | - |
| STB19 | STB | 1998 | H | - |
| STB20 | STB | 1998 | H | - |
| MTB1 | *M. tuberculosis* | 2003 | LAM1 | ST20 |
| MTB2 | *M. tuberculosis* | 2002 | LAM4 | ST60 |
| MTB3 | *M. tuberculosis* | 2002 | LAM4 | ST60 |
| MTB4 | *M. tuberculosis* | 2002 | LAM4 | ST60 |
| MTB5 | *M. tuberculosis* | 2003 | LAM4 | ST60 |
| MTB6 | *M. tuberculosis* | 2002 | LAM4 | ST828 |
| MTB7 | *M. tuberculosis* | 2002 | LAM5 | ST93 |
| MTB8 | *M. tuberculosis* | 2005 | LAM5 | ST93 |
| MTB9 | *M. tuberculosis* | 2002 | LAM9 | ST42 |
| MTB10 | *M. tuberculosis* | 2002 | LAM9 | ST42 |
| MTB11 | *M. tuberculosis* | 2002 | LAM9 | ST42 |
| MTB12 | *M. tuberculosis* | 2003 | LAM9 | ST42 |
| MTB13 | *M. tuberculosis* | 2003 | LAM9 | ST42 |
| MTB14 | *M. tuberculosis* | 2004 | LAM9 | ST42 |
| MTB15 | *M. tuberculosis* | 2004 | LAM9 | ST42 |
| MTB16 | *M. tuberculosis* | 2004 | LAM9 | ST42 |
| MTB17 | *M. tuberculosis* | 2004 | LAM9 | ST42 |
| MTB18 | *M. tuberculosis* | 2005 | LAM9 | ST42 |
| MTB19 | *M. tuberculosis* | 2005 | LAM9 | ST42 |
| MTB20 | *M. tuberculosis* | 2005 | LAM9 | ST42 |
| MTB21 | *M. tuberculosis* | 2004 | LAM9 | ST177 |
| MTB22 | *M. tuberculosis* | 2005 | LAM9 | ST177 |
| MTB23 | *M. tuberculosis* | 2002 | LAM9 | ST398 |
| MTB24 | *M. tuberculosis* | 2005 | LAM9 | ST822 |
| MTB25 | *M. tuberculosis* | 2005 | LAM9 | ST822 |
| MTB26 | *M. tuberculosis* | 2003 | LAM9 | ST1064 |
| MTB27 | *M. tuberculosis* | 2004 | LAM9 | ST1064 |
| MTB28 | *M. tuberculosis* | 2002 | H1 | ST47 |
| MTB29 | *M. tuberculosis* | 2003 | H1 | ST47 |
| MTB30 | *M. tuberculosis* | 2003 | H1 | ST47 |
| MTB31 | *M. tuberculosis* | 2004 | H1 | ST47 |
| MTB32 | *M. tuberculosis* | 2004 | H1 | ST47 |
| MTB33 | *M. tuberculosis* | 2004 | H1 | ST47 |
| MTB34 | *M. tuberculosis* | 2003 | H1 | ST883 |
| MTB35 | *M. tuberculosis* | 2005 | H3 | ST49 |
| MTB36 | *M. tuberculosis* | 2001 | H3 | ST50 |
| MTB37 | *M. tuberculosis* | 2002 | H3 | ST50 |
| MTB38 | *M. tuberculosis* | 2002 | H3 | ST50 |
| MTB39 | *M. tuberculosis* | 2003 | H3 | ST50 |
| MTB40 | *M. tuberculosis* | 2003 | H3 | ST50 |
| MTB41 | *M. tuberculosis* | 2005 | H3 | ST50 |
| MTB42 | *M. tuberculosis* | 2005 | H3 | ST50 |
| MTB43 | *M. tuberculosis* | 2003 | H3 | ST56 |
| MTB44 | *M. tuberculosis* | 2004 | H3 | ST180 |
| MTB45 | *M. tuberculosis* | 2004 | H3 | ST871 |
| MTB46 | *M. tuberculosis* | 2002 | H3 | ST121 |
| MTB47 | *M. tuberculosis* | 2002 | H3 | ST764 |
| MTB48 | *M. tuberculosis* | 2002 | T1 | ST7 |
| MTB49 | *M. tuberculosis* | 2001 | T1 | ST53 |
| MTB50 | *M. tuberculosis* | 2002 | T1 | ST53 |
| MTB51 | *M. tuberculosis* | 2002 | T1 | ST53 |
| MTB52 | *M. tuberculosis* | 2003 | T1 | ST53 |
| MTB53 | *M. tuberculosis* | 2003 | T1 | ST53 |
| MTB54 | *M. tuberculosis* | 2003 | T1 | ST53 |
| MTB55 | *M. tuberculosis* | 2004 | T1 | ST53 |
| MTB56 | *M. tuberculosis* | 2004 | T1 | ST53 |
| MTB57 | *M. tuberculosis* | 2004 | T1 | ST53 |
| MTB58 | *M. tuberculosis* | 2005 | T1 | ST53 |
| MTB59 | *M. tuberculosis* | 2004 | T1 | ST281 |
| MTB60 | *M. tuberculosis* | 2005 | T2 | ST52 |
| MTB61 | *M. tuberculosis* | 2004 | MANU2 | ST54 |
| MTB62 | *M. tuberculosis* | 2004 | S | ST34 |
| MTB63 | *M. tuberculosis* | 2005 | S | ST1536 |

^1^ STB genetic groups were determined by MIRU-VNTR 24 as previously described [19,20].

^2^ Typing of MTB clinical strains was performed by spoligotyping using the method of Kamerbeek et al. (Kamerbeek J, Schouls L, Kolk A, van Agterveld M, van Soolingen D, kuijper S, et al. 1997. Simultaneous detection and strain differentiation of Mycobacterium tuberculosis for diagnosis and epidemiology. J Clin Microbiol 35:907-914.)

Spoligotyping patterns and their corresponding shared types (ST) were defined according to the definitions in the SITVITWEB database (http://www.pasteur-guadeloupe.fr:8081/SITVIT_ONLINE/).
