# Supplementary material for: Evolutionary Trends of the Transposase-Encoding Open Reading Frames A and B (orfA and orfB) of the Mycobacterial IS6110 Insertion Sequence
Source: PLoS One. 2015 Jun 18;10(6):e0130161. doi: 10.1371/journal.pone.0130161 (PMC4473070; doi:10.1371/journal.pone.0130161)
Supplement: S2 Table — (DOCX) [file pone.0130161.s002.docx]

S2 Table. The best-fit model of nucleotide substitution as determined by jModelTest 2 [51].

|  | **model** | **-lnL** |
| --- | --- | --- |
| *orfA*-STB+MTB | HKY | 613.9731 |
| *orfA*-MTB | HKY | 518.9148 |
| *orfA*-STB | HKY | 528.7957 |
| *orfB*-STB+MTB | TPM3uf | 1716.111 |
| *orfB*-MTB | HKY | 518.9148 |
| *orfB*-STB | TPM3uf | 1448.0068 |
